# High-entropy thermal-stiffening hydrogels with fast switching dynamics

**DOI:** 10.1093/nsr/nwaf072

**Published:** 2025-02-27

**Authors:** Li Li, Baohu Wu, Shengtong Sun, Peiyi Wu

**Affiliations:** State Key Laboratory of Advanced Fiber Materials, College of Chemistry and Chemical Engineering & Center for Advanced Low-Dimension Materials, Donghua University, Shanghai 201620, China; Jülich Centre for Neutron Science (JCNS) at Heinz Maier-Leibnitz Zentrum (MLZ) Forschungszentrum Jülich, Garching 85748, Germany; State Key Laboratory of Advanced Fiber Materials, College of Chemistry and Chemical Engineering & Center for Advanced Low-Dimension Materials, Donghua University, Shanghai 201620, China; State Key Laboratory of Advanced Fiber Materials, College of Chemistry and Chemical Engineering & Center for Advanced Low-Dimension Materials, Donghua University, Shanghai 201620, China

**Keywords:** hydrogels, responsive materials, phase separation, modulus control, toughening, impact-resistant

## Abstract

Thermal-stiffening hydrogels exhibit a dramatic soft-to-stiff transition upon heating, making them ideal candidates for temperature-triggered self-protection and shape memory applications. However, their practical use is still hampered by a slow recovery process (generally >30 min) during cooling, attributed to sluggish mass diffusion and delayed phase dissolution. Herein, we present a high-entropy phase separation design to significantly accelerate the recovery dynamics of these materials. We demonstrate this concept using a thermal-stiffening poly(calcium acrylate)-based copolymer hydrogel by incorporating hydrophilic units. Mechanistically, the hydrophilic units disrupt the dense packing of thermal-stiffening clusters, creating a high-entropy topological structure with a low energy barrier for rapid mass diffusion. This approach retains the impressive thermal-stiffening response with a 760-fold increase in storage modulus, while dramatically reducing the characteristic recovery time to merely 28 s. We anticipate this high-entropy strategy to be broadly applicable in designing modulus-adaptive materials with fast switching dynamics.

## Teaser

This work puts forward a high-entropy phase separation concept to promote the recovery dynamics of thermal-stiffening hydrogels without compromising their exceptional stiffening response.

## INTRODUCTION

Materials usually soften with increasing temperature, because the binding physical interactions are generally enthalpy-dominated and become less stable at elevated temperatures [1–4]. To address the challenge of heat-induced mechanical degradation, thermal-stiffening materials have garnered great attention in recent years. Polyacrylic acid (PAA) hydrogels physically crosslinked by Ca^2+^ offer a prominent example of such materials. Unlike conventional lower-critical-solution-temperature-(LCST)-type polymer gels that exhibit significant volume change and modest stiffness increase (generally <20 times) [5–9], Ca^2+^-coordinated PAA hydrogels undergo an isochoric, dramatic transition from a rubbery or viscous state to a glassy state upon heating, resulting in a sharp and reversible increase in stiffness by up to 13 000 times [10–12]. This unique property has enabled a diverse range of applications, including impact protection [10], smart fabrics [11,13], heat absorption [10,13], shape memory [14–16], actuation [17], anti-counterfeiting [18], ionic switching [19] and adaptive lubrication [20,21].

**Figure 1.**
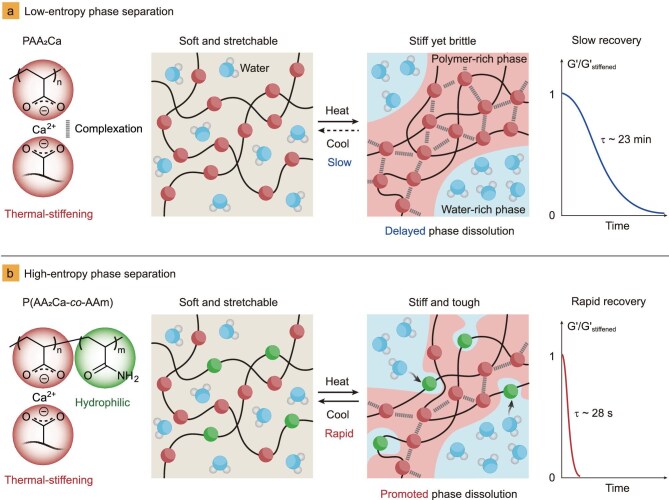
Schematic high-entropy phase separation for accelerating the switching dynamics of thermal-stiffening hydrogels. (a) Conventional low-entropy phase separation: upon heating, the neat PAA_2_Ca hydrogel undergoes a dramatic transition from a soft and stretchable state to a stiff yet brittle state. However, due to the delayed dissolution of the low-entropy glassy phase, the recovery upon cooling is rather slow, with a long characteristic time of ∼23 min. (b) High-entropy phase separation: the P(AA_2_Ca-*co*-AAm) copolymer hydrogel also exhibits a dramatic soft-to-stiff transition upon heating. However, the resulting stiffened hydrogel is significantly tougher than the neat PAA_2_Ca hydrogel. In addition, the phase-separated high-entropy topological structure promotes rapid phase dissolution, leading to ultra-rapid recovery with a characteristic time of merely 28 s.

Despite the impressive ultra-rapid stiffening upon heating (within seconds), the hydrogel usually exhibits a significant drawback: slow modulus recovery (softening) that can take over 30 min upon cooling. This disparity in stiffening-recovery rates may be advantageous for designing shape memory devices with programmable recovery time [14], but hinders applications that require rapid modulus changes, such as soft armors and actuators. Thermodynamically, the sluggish recovery stems from the slow mass diffusion during the spinodal decomposition, a spontaneous phase separation process. As illustrated in Fig. 1a, heat triggers the decomposition of the thermal-stiffening hydrogel into polymer-rich and water-rich phases. Upon exceeding the critical stiffening temperature (i.e. Berghmans’ point for LCST-type polymers) [22,23], the continuous polymer-rich phase rapidly vitrifies, forming a stiff network with restricted chain mobility. Conversely, the recovery upon cooling relies on the slow hydration and dissolution of the glassy polymer-rich phase, leading to the extended softening time. Notably, this challenge is unique to phase-separation-induced thermal-stiffening hydrogels, while conventional thermal-softening materials often experience rapid modulus changes dominated by heat transfer [14,24]. Incorporation of α-methyl groups to elevate the stiffening temperature may expedite the recovery process; however, this approach often comes at the cost of the stiffening response, resulting in a diminished degree of phase separation [25]. So far, it remains a significant challenge to accelerate the recovery dynamics of thermal-stiffening hydrogels without compromising their exceptional stiffening response.

In this paper, we introduce a novel high-entropy phase-separation design to accelerate the recovery of thermal-stiffening hydrogels. Inspired by high-entropy materials for overcoming material challenges like the strength-toughness trade-off [26–30], we anticipate that enhancing the topological entropy of the phase-separated structure shall significantly promote phase dissolution and mass diffusion in thermal-stiffening hydrogels. For phase-separated systems, a high entropy generally correlates with a high mixing level between disparate phases at a constant total volume fraction. As such, increased mixing entropy manifests as a decrease in phase domain size and an increase in the interfacial area (or topological fractal dimension) of phase-separated morphologies [26,31,32]. To validate this concept, we synthesized a series of thermal-stiffening hydrogels by copolymerizing calcium acrylate (AA_2_Ca, the thermal-stiffening unit) and acrylamide (AAm, the hydrophilic unit) (Fig. 1b). The AAm units improve compatibility between the separated polymer-rich and water-rich phases, arresting microphase separation and increasing topological entropy at the nanoscale [31,33]. This effect greatly lowers the energy barrier between the two separated phases, thus facilitating the dissolution of the glassy phase during cooling. Our optimized copolymer hydrogel exhibits a remarkably rapid recovery with a short characteristic time (τ) of merely 28 s, significantly faster than the neat PAA_2_Ca hydrogel (τ ∼ 23 min) and previous thermal-stiffening hydrogels. We attribute this accelerated recovery to the loose assembly of primary thermal-stiffening clusters separated by the hydrophilic units, supporting the high-entropy topological design. Importantly, the hydrogel retains its excellent thermal-stiffening behavior, transitioning from an ultra-soft and stretchable state at room temperature to a stiff and tough state at high temperatures, with a remarkable 760-fold increase in storage modulus.

## RESULTS

### Preparation and mechanical properties

Thermal-stiffening P(AA_2_Ca-*co*-AAm) copolymer hydrogels were synthesized by the direct copolymerization of AA_2_Ca and AAm monomers in an aqueous precursor at room temperature, without the use of chemical crosslinkers. The molar content of AAm relative to AA_2_Ca varied from 0% to 30% to optimize the hydrogel's mechanical properties and temperature-triggered stiffening/recovery behavior. Notably, this is the first time that AA_2_Ca monomer has been directly employed for the synthesis of dramatic thermal-stiffening hydrogels. Previous approaches mainly involved soaking pre-prepared PAA hydrogels in calcium acetate aqueous solution or hybridizing PAA chains with amorphous calcium carbonate (ACC) clusters, which are either time-consuming or lack precise control over the polymer network structure [10,11,14,16]. As we previously reported, the thermal-stiffening behavior originates from the complexation between Ca^2+^ and COO^−^, leading to a reduced solubility in water at high temperatures [11,34]. One key advantage of AA_2_Ca monomer lies in its ability to create various PAA_2_Ca-based copolymers. These copolymers incorporate associative COO^−^ groups, essentially acting as supramolecular ‘sticky’ polymers, which offers vast possibilities for engineering adaptive soft materials [35–40].

**Figure 2.**
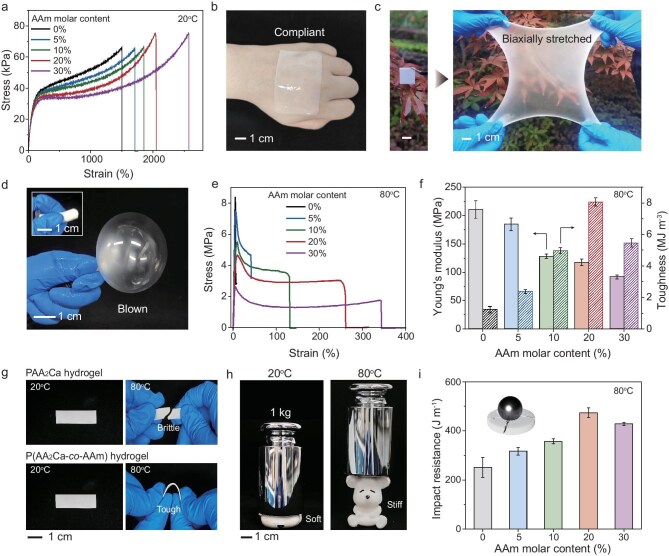
Mechanical properties of thermal-stiffening P(AA_2_Ca-*co*-AAm) hydrogels. (a) Tensile stress-strain curves of the copolymer hydrogels with increasing AAm molar contents at room temperature. (b) Photo of the copolymer hydrogel with 20% AAm on a human hand demonstrating its good compliance. (c, d) The copolymer hydrogel with 20% AAm can be either biaxially stretched or blown with good elastic recovery. (e) Tensile stress-strain curves of P(AA_2_Ca-*co*-AAm) hydrogels with increasing AAm molar contents at 80°C. (f) Corresponding Young's moduli and toughness. (g) Bending tests of the stiffened PAA_2_Ca and P(AA_2_Ca-*co*-AAm) hydrogels. (h) The copolymer hydrogel with 20% AAm has distinct load-bearing capacities at 20 and 80°C. (i) Impact resistance of the stiffened copolymer hydrogels with increasing AAm molar contents.

All the P(AA_2_Ca-*co*-AAm) copolymer hydrogels swollen in water appeared opaque, yet were highly soft and stretchable at room temperature (20°C, Fig. S1). The opacity resulted from the spontaneous phase separation of Ca^2+^-crosslinked polymer chains, which have limited solubility in water. With increasing AAm contents, the hydrogels became more stretchable, experiencing a slight decrease in modulus (∼10 kPa, Fig. 2a). This is attributed to the reduced physical crosslinking density caused by the incorporation of hydrophilic AAm units. Consequently, both the water content and the mobility of water molecules within the hydrogel increased with increasing AAm contents (Figs S2, S3). The representative hydrogel with 20% AAm exhibited exceptional compliance, readily conforming to curved surfaces like a human hand (Fig. 2b). Moreover, it could be stretched over 20 times its original length and readily recovered from both stretching and inflation (Fig. 2c and d, Fig. S4). This excellent elastic recovery stems from the high chain entanglement which acts as topological crosslinks for entropic elasticity.

Upon immersion in hot water (80°C), all copolymer hydrogels exhibited immediate stiffening within seconds with a minimal volume change. The selection of 80°C as the heating temperature was predicated on the need to ensure complete phase separation, while avoiding a possible dehydration issue, which could impede accurate material characterizations at more elevated temperatures. At the stiffened state, the neat PAA_2_Ca hydrogel displayed the highest stiffness, reaching a Young's modulus of 210 MPa (Fig. 2e and f). However, its brittleness was also evident, as demonstrated by a toughness value of only 1.3 MJ m^−3^ and the bending test (Fig. 2f and g). Increasing the AAm content progressively reduced the modulus in the stiffened state while enhancing the elongation of the hydrogel. This is understandable since the hydrophilic AAm units do not contribute to the thermal-stiffening response but rather plasticize the rigid polymer phase at high temperatures. The optimal balance between stiffness and toughness was achieved for the hydrogel with 20% AAm, exhibiting a Young's modulus of 118 MPa, an elongation of 260% and a toughness of 8.1 MJ m^−3^. Upon bending, the stiffened copolymer hydrogel deformed plastically without fracture, showcasing its high toughness (Fig. 2g).

Moreover, the remarkable soft-to-stiff transition can be visually demonstrated by a load-bearing test (Fig. 2h). At room temperature, the copolymer hydrogel displayed extreme softness and easy deformation. However, in the stiffened state upon heating, it could support a substantial weight of 1 kg. Further increasing AAm content to 40% and 50% resulted in a significantly weakened thermal-stiffening response and possible volume contraction (Fig. S5), which will not be discussed in this paper. We also conducted drop-ball impact tests to evaluate the impact resistance of the stiffened hydrogels with varying AAm contents [41–43]. As expected, the optimal copolymer hydrogel with 20% AAm displayed also a highest impact resistance of 474 J m^−1^, aligning with its exceptional toughness (Fig. 2i).

### Recovery dynamics of thermal-stiffening hydrogels

**Figure 3.**
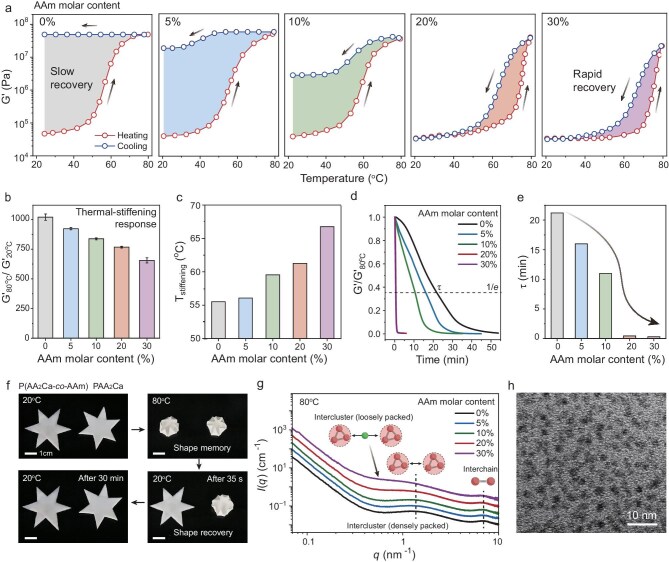
Recovery dynamics of thermal-stiffening P(AA_2_Ca-*co*-AAm) hydrogels. (a) Rheological heating and cooling curves of the copolymer hydrogels with increasing AAm molar contents (scanning rate: 4°C min^−1^). (b) Thermal-stiffening response quantified by the ratio of their storage moduli at 80°C and 20°C. (c) Stiffening temperatures determined from the tan δ peaks during heating. (d) Time-dependent recovery of the storage moduli. All the hydrogels were initially cured at 80°C for 30 min and subsequently immersed in room-temperature water for monitoring. (e) Corresponding characteristic recovery times. (f) Photos visualizing the shape recovery process of the stiffened PAA_2_Ca hydrogel and the P(AA_2_Ca-*co*-AAm) hydrogel with 20% AAm. (g) Stacked SAXS profiles of the copolymer hydrogels with increasing AAm molar contents at 80°C. (h) TEM image of the stiffened copolymer hydrogel with 20% AAm (quenched at 80°C and then freeze-dried).

To comprehensively investigate the thermal-stiffening and recovery dynamics of the hydrogels, we performed temperature-sweep rheological measurements at a constant heating and cooling rate of 4°C min^−1^ (Fig. 3a). All hydrogels exhibited a significant increase in their storage moduli (G′) at ∼60°C, demonstrating a pronounced thermal-stiffening behavior. The neat PAA_2_Ca hydrogel displayed the most pronounced stiffening response, with a remarkable 1016-fold increase in storage modulus upon heating from 20 to 80°C (Fig. 3b). Incorporating AAm units into the copolymer progressively reduced this stiffening response, but even the copolymer hydrogels with 20% and 30% AAm still exhibited an increase in their storage moduli by 760 and 640 times, respectively. This suggests that a well-balanced amount of AAm did not significantly disrupt the formation of a continuous glassy phase in the stiffened state. Moreover, the stiffening temperature showed an upward trend with increasing AAm contents (from 56.2°C for the neat PAA_2_Ca hydrogel to 62.3°C and 66.1°C for the copolymer hydrogels with 20% and 30% AAm, respectively), determined by both rheological tan δ peaks and calorimetric measurements (Fig. 3c, Figs S6 and S7).

Crucially, the recovery behavior upon cooling differed significantly among these samples. The neat PAA_2_Ca hydrogel exhibited minimal recovery within the experimental timescale (Fig. 3a), which aligns with observations in previously reported PAA/calcium acetate and ACC-PAA hydrogels [10,11,14]. Incorporating AAm units significantly reduced the hysteresis, leading to faster recovery dynamics. To quantify the recovery process, we conducted time-sweep rheological measurements. This involved immersing the stiffened hydrogels (cured at 80°C for 30 min) in room-temperature water and monitoring the change in modulus over time (Fig. 3d). All hydrogels exhibited a decrease in modulus with time, but at different rates as reflected by their characteristic times (τ, the time it takes for the modulus to decrease to 1/e of its original value). The neat PAA_2_Ca hydrogel displayed a slow recovery process with a lengthy characteristic time of 23 min. However, for copolymer hydrogels with 20% and 30% AAm, the characteristic time was dramatically reduced to 28 and 22 s, respectively (Fig. 3e). Substitution of AAm with alternative hydrophilic monomers, such as acrylic acid (AA), hydroxyethyl acrylate (HEA) and *N,N*-dimethylacrylamide (DMA), also led to markedly reduced recovery times, underscoring the broad applicability of our methodology (Fig. S8).

Increasing the curing time remarkably prolonged the recovery time of PAA_2_Ca hydrogel, yet did not apparently affect the fast recovery behavior of the copolymer hydrogel with 20% AAm (Fig. S9). This suggests that incorporating AAm content significantly altered the topological structure of the stiffened polymer phase. The comparison of PAA_2_Ca and copolymer hydrogels visually highlighted the significantly faster shape recovery upon cooling achieved through the incorporation of AAm units (Fig. 3f). As a comparison, the characteristic recovery times of conventional PAA/calcium acetate and ACC-PAA hydrogels were measured to be 77 and 31 min, respectively (Fig. S10), also considerably longer than those observed for the present P(AA_2_Ca-*co*-AAm) copolymer hydrogels.

To gain insights into the internal structural changes upon incorporating AAm units, we employed small-angle X-ray scattering (SAXS) and very-small-angle neutron scattering (VSANS) measurements. At room temperature (20°C), the scattering patterns of all hydrogels exhibited similar characteristics for a chain-like gel structure (Fig. S11). However, at a high temperature (80°C), the SAXS data revealed distinct information about clustering within the glassy polymer phase (Fig. 3g). All samples displayed a characteristic interchain distance of ∼0.9 nm at the high *q* regime (*q* = 7 nm^−1^), which was absent at room temperature. This arises from the further dehydration of PAA_2_Ca, leading to collapsed polymer chains. Additionally, in the intermediate *q* regime (0.5 nm^−1^ < *q* < 1.5 nm^−1^), a scattering peak emerged, indicating the presence of clusters with an approximate distance of 4.2 nm. This suggests that the glassy phase is actually formed by the close packing of dehydrated PAA_2_Ca clusters with sizes smaller than 2 nm (radius of gyration, *R*_g_ ∼0.8 nm). The clustering morphology can be further confirmed by transmission electron microscopy (TEM) observation showing a mean cluster size of 1.3 nm in the dried state (Fig. 3h). We attribute these clusters to the ionic complexation between locally concentrated Ca^2+^ and COO^−^ groups. This mode of cluster formation is reminiscent of amorphous calcium carbonate clusters, a structural motif commonly identified in mineralization systems [11,44–46].

Importantly, as the AAm content increased to 20%, the SAXS scattering intensity in the intermediate *q* region formed a plateau, suggesting a more random arrangement of clusters. Further increasing the AAm content to 30% led to a rise in this plateau, which eventually merged with the low *q* scattering curve. This indicates that incorporating AAm units progressively separated the dehydrated PAA_2_Ca clusters, leading to a larger distance between loosely packed clusters. This observation well supports our hypothesis that incorporating hydrophilic units can indeed increase the topological entropy of the separated phase. Notably, such an increase in topological entropy was primarily observed at the nanoscale. VSANS measurements revealed a very similar macroscopic phase-separated structure across all the hydrogels at 80°C, with an average domain size of ∼11 μm (equivalent to 2.58 *R*_g_, *R*_g_ ∼4.4 μm; Fig. S12).

Collectively, all the above characterizations point to a critical AAm content of 20%, marking the formation of phase-separated high-entropy topological structure. Such a unique structure is believed to possess a low-energy barrier and an expanded interfacial area between the collapsed polymer-rich phase and surrounding water. This accounts for the observed fast recovery dynamics by promoting phase dissolution during cooling. Notably, the formation of a high-entropy topological structure does not significantly compromise the remarkable thermal-stiffening response of the hydrogel. In fact, it even contributes to a remarkable increase in toughness in the stiffened state. Unless otherwise stated, all the copolymer hydrogels hereafter refer to the sample with 20% AAm content.

### Mechanism discussion for high-entropy thermal stiffening

**Figure 4.**
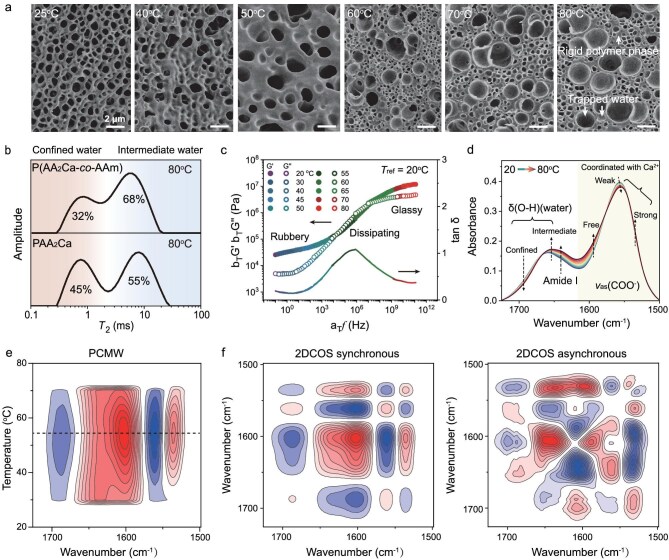
Mechanism analysis for high-entropy phase separation. (a) SEM images of lyophilized P(AA_2_Ca-*co*-AAm) hydrogels with 20% AAm, which were preheated to different temperatures and then quenched using liquid nitrogen. (b) Low-field ^1^H NMR spectra of PAA_2_Ca and P(AA_2_Ca-*co*-AAm) hydrogels at 80°C. (c) Reverse time-temperature superposition rheological curves of the copolymer hydrogel with 20% AAm at a reference temperature of 20°C. (d) Temperature-variable FTIR spectra of the copolymer hydrogel from 20 to 80°C (interval: 4°C). In the 2D spectra, red colors represent positive spectral intensities, while blue colors represent negative ones. (e, f) PCMW spectrum and 2DCOS synchronous and asynchronous spectra generated from (d).

To get a deeper understanding of the high-entropy thermal-stiffening behavior, we first monitored the heat-induced phase separation process by scanning electron microscopy (SEM) observation. The samples were prepared by quenching the evolving phase structure at different temperatures using liquid nitrogen, and then freeze-drying. As shown in Fig. 4a, the sample at room temperature displayed a relatively uniform, porous structure, corresponding to the soft polymer phase. Rising temperature made polymer chains collapse further, and above the stiffening temperature (∼60°C), structural inhomogeneity became evident. Dehydrated polymer chains were frozen in the glassy state, forming a continuous and rigid polymer framework. The collapse of the gel structure and the formation of dehydrated clusters embedded within the glassy gel can also be clearly observed in the temperature-dependent SAXS curves (Fig. S13). Meanwhile, the water released during phase separation became trapped within the newly formed nano- and micropores. This corresponds to a microsyneresis procedure, which explains the macroscopic observations of constant volume and weight during phase separation (with a solid content of ∼36 wt%; Fig. S14). Furthermore, the phase-separated morphologies of P(AA_2_Ca-*co*-AAm) copolymer hydrogels exhibited no discernible variation across different AAm contents (Fig. S15). This consistency reinforces the conclusion that the high-entropy structure primarily originates from the nanoassembly of thermal-stiffening clusters, rather than being evident at the macroscopic scale.

To understand how the mobility of water molecules changes within the hydrogel upon heating, we employed temperature-variable low-field ^1^H nuclear magnetic resonance (NMR) spectroscopy. In this technique, the spin-spin relaxation time (*T*_2_) reflects the freedom of hydrogen atoms, and in this case, primarily water activity [11,41,47]. Similar to SEM observation, the initially loosely confined water molecules at low temperatures split into two distinct states upon heating (Fig. 4b, Fig. S16). One state represents the water molecules strongly confined within the glassy polymer phase with a *T*_2_ of ∼0.8 ms, and the other one represents the water molecules trapped within the nano- and micropores, exhibiting a *T*_2_ of ∼7 ms. We compared the low-field ^1^H NMR spectra of the neat PAA_2_Ca and copolymer hydrogels at 80°C. Notably, the copolymer hydrogel displayed a significantly higher proportion of intermediate water (68%) than that of the neat PAA_2_Ca hydrogel (55%). This observation suggests that the high-entropy topological structure of the copolymer hydrogel provided more expanded phase boundaries, capable of accommodating a larger amount of intermediate water. The embedded intermediate water in the glassy phase likely plays a crucial role in promoting phase dissolution during the cooling process, facilitating the recovery of the hydrogel to its original state.

Time-temperature superposition rheology was further utilized to study the viscoelasticity of the copolymer hydrogel. Unlike conventional thermal-softening materials [38,47,48], the frequency-sweep rheological curves of both the neat PAA_2_Ca and copolymer hydrogels could be superposed reversely with longer timescales at lower temperatures (reference temperature = 20°C; Fig. 4c, Fig. S17). As the frequency or temperature increased, the copolymer hydrogel underwent three distinct rheological states: rubbery, dissipating and glassy. At the experimental quasi-static timescale (∼0.1 Hz), the hydrogel exhibited rubbery behavior with a low loss factor (tan δ = 0.19), indicating a highly elastic response. The number-average molar mass of the entanglement strands of the copolymer hydrogel was estimated to be 8.2 × 10^4^ g mol^−1^ using the minimum tan δ method (Fig. S18). This value is sufficiently high to promote chain entanglement and contribute to the high entropic elasticity at room temperature. Elevating the temperature to the stiffening temperature (∼60°C) forced the copolymer hydrogel into the dissipating regime (also known as the glass transition regime with a tan δ peak). In this state, highly sticky physical complexation between Ca^2+^ and COO^−^ began to play a dominant role in controlling the whole chain dynamics. Further increasing temperature effectively froze the segmental motion of polymer chains, leading to vitrification and the dramatic stiffening of the hydrogel. It is noteworthy that in the glassy state, the tan δ value of the copolymer hydrogel (∼0.4) is higher compared to the neat PAA_2_Ca hydrogel (tan δ ∼0.3). This accords with the formation of a high-entropy topological structure and the observed higher plasticity and toughness in the stiffened copolymer hydrogel.

Finally, to clarify the interaction changes at the molecular level, temperature-variable Fourier transform infrared (FTIR) spectra in combination with perturbation-correlation moving window (PCMW) and 2D correlation spectra (2DCOS) were recorded and analyzed [49,50]. We focused on the spectral region from 1725 to 1500 cm^−1^, which contains the vibrational information of δ(O-H) of water, amide I of PAAm and *v*_as_(COO^−^) of PAA_2_Ca. As shown in Fig. 4d, as temperature increased, the spectral intensity of δ(O-H) assigned to confined water decreased while that to intermediate water increased, suggesting their mutual transformation. Additionally, the spectral intensity of *v*_as_(COO^−^) assigned to weakly coordinated COO^−^ groups with Ca^2+^ decreased, while those to free and strongly coordinated COO^−^ groups increased. This observation suggests that the dehydration of PAA_2_Ca during heating led to collapsed polymer chains with a higher Ca^2+^ coordination number [11]. The PCMW technique, based on the temperature-variable FTIR spectra, calculated the transition temperature for different groups to all be ∼56°C (Fig. 4e), consistent with rheological and differential scanning calorimetry (DSC) measurements.

2DCOS spectra were further generated to determine the thermal-responsive sequence of different groups during the stiffening process [37,49]. Based on Noda's judging rule and the signs of cross-peaks in the synchronous and asynchronous spectra (Fig. 4f, Table S1), the following sequential order of events during heating was established (→ means prior to or earlier than): δ(O-H) (intermediate water, 1650 cm^−1^) → *v*(C=O) (amide I, PAAm, 1641 cm^−1^) → δ(O-H) (confined water, 1683 cm^−1^) → *v*_as_(COO^−^) (free, 1596 cm^−1^) → *v*_as_(COO^−^) (weakly coordinated with Ca^2+^, 1562 cm^−1^) → *v*_as_(COO^−^) (strongly coordinated with Ca^2+^, 1538 cm^−1^). The earliest response of δ(O-H) suggests that the thermal-stiffening process of the copolymer hydrogel was driven by the transformation between confined water and intermediate water (i.e. water redistribution within the hydrogel). Furthermore, the second-place sequence of amide I from PAAm underlines the significant role played by the hydrophilic units, which actively influence the phase separation process, promoting the formation of a high-entropy topological structure.

## CONCLUSION

In summary, this paper presents a novel thermal-stiffening hydrogel featuring a stiffened high-entropy structure, capable of achieving both significant stiffness modulation and rapid switching dynamics. We highlight the design of a high-entropy topological structure by incorporating hydrophilic units into the thermal-stiffening PAA_2_Ca hydrogel, which improves the compatibility between separated phases and suppresses microphase separation. In its stiffened state, the resulting copolymer hydrogel exhibits a low energy barrier and an expanded interfacial area, facilitating rapid mass diffusion. Consequently, the glassy polymer phase readily dissolves upon cooling, enabling fast recovery with a short characteristic time of only 28 s—significantly outperforming previous thermal-stiffening hydrogels. At the nanoscale, the high-entropy topological structure is formed by the loose packing of thermal-stiffening clusters separated by the incorporated hydrophilic units. At the molecular level, this process is driven by water redistribution mediated by the hydrophilic units. We believe that this high-entropy phase separation design offers a promising strategy to control the responsive behavior of modulus-adaptive materials. By leveraging this approach, we may develop fast-recovery thermal-stiffening hydrogels with potential applications in soft armor and soft actuators requiring rapid modulus control.

## MATERIALS AND METHODS

### Preparation of calcium acrylate (AA_2_Ca) precursor

The AA_2_Ca precursor was synthesized by first dissolving 42 mL of AA (0.6 mol) in 100 mL of deionized water. Under stirring, equimolar Ca(OH)_2_ powder (22.23 g, 0.3 mol) was then slowly added to the AA solution. The mixture was subsequently stirred continuously for another 1 h in a 60°C water bath to facilitate the reaction. Finally, the mixture was filtered and centrifuged to remove any unreacted impurities, yielding a transparent and colorless AA_2_Ca aqueous precursor.

### Preparation of P(AA_2_Ca-*co*-AAm) hydrogels

0.1 mol% α-ketoglutaric acid (photo-initiator, relative to AA_2_Ca) was first dissolved in the AA_2_Ca precursor. Then, AAm was added in varying amounts (0%, 5%, 10%, 20%, 30%, 40% and 50% molar content relative to AA_2_Ca). A 60°C water bath was used to ensure complete dissolution of all components. After degassing to remove any trapped air bubbles, the copolymer precursor solution was injected into a sandwiched glass mold and polymerized under UV irradiation for 30 min at room temperature. The resulting opaque hydrogels were initially submerged in 4°C water for 2 days to expedite swelling, followed by immersion in 20°C water until an equilibrium was reached prior to use.

### Rheological measurements

The rheological behavior of hydrogels was investigated on a Thermo Scientific HAAKE MARS 60 modular advanced rheometer in the oscillation mode with a parallel-plate geometry (plate diameter: 8 mm). For temperature-sweep measurements, the frequency and shear strain were fixed to 0.1 Hz and 0.07%, respectively. A heating-cooling cycle from 20 to 80°C was applied with a scanning rate of 4°C min^−1^ without pause. For time-sweep measurements, after the hydrogels were initially cured at 80°C for 30 min and then immersed in room-temperature water, the storage modulus was monitored with time at a frequency of 0.1 Hz and a shear strain of 0.07%. For frequency-sweep measurements, the shear strain was fixed to 0.07%, and temperature varied to collect rheological data for time-temperature superposition.

### Small-angle X-ray scattering

SAXS experiments were performed using a laboratory-based SAXS beamline, KWS-X (XENOCS XUESS 3.0 XL) at JCNS-MLZ, Garching, Germany. The MetalJet X-ray source (Excillum D2+) with a liquid metal anode was operated at 70 kV and 3.57 mA, emitting Ga-Kα radiation with a wavelength of λ = 1.314 Å. Samples were measured in sealed capillaries (inner diameter: 2 mm) on a temperature-controlled stage. The sample-to-detector distances varied from 0.5 to 1.7 m. The SAXS patterns were normalized to an absolute scale and azimuthally averaged to obtain the intensity profiles, and the solvent background was subtracted.

## Supplementary Material

nwaf072_Supplemental_File
