# Supplementary material for: High-entropy thermal-stiffening hydrogels with fast switching dynamics
Source: Natl Sci Rev. 2025 Feb 27;12(4):nwaf072. doi: 10.1093/nsr/nwaf072 (PMC11929134; doi:10.1093/nsr/nwaf072)
Supplement: nwaf072_Supplemental_File [file nwaf072_supplemental_file.pdf]

Supplemental Data

High-entropy thermal-stiffening hydrogels with fast switching dynamics

Li Li[1], Baohu Wu[2], Shengtong Sun[1,*], and Peiyi Wu[1,*]

[1]State Key Laboratory of Advanced Fiber Materials, College of Chemistry and Chemical Engineering & Center for Advanced Low-dimension Materials, Donghua University, Shanghai 201620, China;

[2]Jülich Centre for Neutron Science (JCNS) at Heinz Maier-Leibnitz Zentrum (MLZ) Forschungszentrum Jülich, Garching 85748, Germany

***Corresponding authors.** E-mail: shengtongsun@dhu.edu.cn; wupeiyi@dhu.edu.cn

Supplemental Figures and Tables

Extended Experimental

Fig. S1. Photos of P(AA_2Ca-*co*-AAm) hydrogels

Fig. S2. Water contents of P(AA_2Ca-*co*-AAm) hydrogels

Fig. S3. Low-field ^{1}H NMR spectra of P(AA_2Ca-*co*-AAm) hydrogels

Fig. S4. Load-unloading and secondary loading curves of P(AA_2Ca-*co*-AAm) hydrogel

Fig. S5. Stress-strain curves and photos of P(AA_2Ca-*co*-AAm) hydrogels with 40-50% AAm

Fig. S6. Temperature-sweep rheological and tan δ curves of P(AA_2Ca-*co*-AAm) hydrogels

Fig. S7. DSC heating curves of P(AA_2Ca-*co*-AAm) hydrogels

Fig. S8. Recovery of P(AA_2Ca-*co*-AA), P(AA_2Ca-*co*-HEA), and P(AA_2Ca-*co*-DMA) hydrogels

Fig. S9. Recovery of PAA_2Ca and P(AA_2Ca-*co*-AAm) hydrogels cured for various durations

Fig. S10. Recovery of PAA/calcium acetate and ACC-PAA hydrogels

Fig. S11. SAXS profiles of P(AA_2Ca-*co*-AAm) hydrogels at 20 °C

Fig. S12. VSANS profiles of P(AA_2Ca-*co*-AAm) hydrogels at 80 °C

Fig. S13. Temperature-dependent SAXS curves of P(AA_2Ca-*co*-AAm) hydrogel

Fig. S14. Solid contents of P(AA_2Ca-*co*-AAm) hydrogel at different temperatures

Fig. S15. SEM images of lyophilized P(AA_2Ca-*co*-AAm) hydrogels with different AAm contents

Fig. S16. Temperature-variable low-field ^{1}H NMR spectra

Fig. S17. Time-temperature superposition rheological curves of neat PAA_2Ca hydrogel

Fig. S18. The minimum tan δ method for determining entanglement plateau modulus

Table S1. Multiplication results of the signs in 2DCOS synchronous and asynchronous spectra

References

Extended Experimental

Materials

Acrylic acid (AA, Adamas), calcium hydroxide ($Ca(OH)_2$, Sinopharm Chemical Reagent), acrylamide (AAm, General-reagent), hydroxyethyl acrylate (HEA, Adamas), *N*,*N*-dimethylacrylamide (DMA, Aladdin), α-ketoglutaric acid (Aladdin), polyacrylic acid (PAA, $M_w \approx$ 100,000 g mol^{-1}, 35 wt% in H_2O, Sigma-Aldrich), calcium acetate (Sinopharm Chemical Reagent), sodium carbonate (Na_2CO_3, Adamas), calcium chloride ($CaCl_2$, Sinopharm Chemical Reagent), *N,N'*-methylenebisacrylamide (MBAA, Sigma-Aldrich). AA, HEA, and DMA were purified by passing through a basic alumina-filled column to remove inhibitors before use.

Preparation of P(AA_2Ca-*co*-AA), P(AA_2Ca-*co*-HEA), and P(AA_2Ca-*co*-DMA) hydrogels

P(AA_2Ca-*co*-AA), P(AA_2Ca-*co*-HEA), and P(AA_2Ca-*co*-DMA) hydrogels were prepared using the same procedure with P(AA_2Ca-*co*-AAm) hydrogel, and only the comonomer was varied to AA, HEA, and DMA (20 mol% relative to AA_2Ca), respectively.

Preparation of PAA/calcium acetate hydrogel

The hydrogel precursor was prepared by mixing 3 M AA aqueous solution with 1 mol% MBAA (crosslinker) and 0.1 mol% α-ketoglutaric acid (photo-initiator). The precursor was then injected into a sandwich glass mold and polymerized under UV irradiation for 60 min at room temperature. The resulting hydrogel was finally treated by immersion in a 250 mM calcium acetate solution for one week.

Preparation of ACC-PAA hydrogel

Equal volume of 0.1 M Na_2CO_3 solution was slowly added to a vigorously stirred mixture containing 0.1 M PAA (concentration refers to the repeating unit) and 0.1 M $CaCl_2$. This resulted

in the gradual formation of a white, paste-like precipitate around the stirring magnet. After continuous stirring for another 1 h, the ACC-PAA hydrogel was collected and washed with deionized water for further purification.

Characterizations

SEM images were taken on a field-emission scanning electron microscope (SEM, Hitachi SU8230). For sample preparation, pre-heated hydrogels at different temperatures for 30 min were immediately quenched in liquid nitrogen and then freeze-dried. DSC was performed on TA DSC250 scanning from -1 to 80 ºC at a heating rate of 5 ºC min^{-1} under nitrogen flow. TEM image was acquired on a JEOL JEM-2100F transmission electron microscope at 200 kV. The sample for TEM was prepared by sonicating the freeze-dried hydrogel (quenched at 80 ºC) in ethanol to create a dispersion, which was then deposited on a copper grid.

Tensile measurements

Tensile stress-strain curves of the hydrogels were recorded on a universal mechanical test machine (UTM2103, Shenzhen Suns technology). For tensile tests at high temperatures, a self-made water jacket was installed on the bottom clamp, and hot water (~80 ºC) was poured into the jacket to ensure the hydrogel remained immersed in the hot water throughout the stretching process. The tensile strain rate was set to 0.02 s^{-1}.

Drop-ball impact tests

To assess the impact resistance of the hydrogels, a drop-ball test method was employed. Hydrogel films with a uniform thickness of 1 mm were positioned over a table containing a central hole. Steel balls with a known weight of 72 g were dropped freely onto the samples from a specific height. The impact resistance was then calculated by dividing the critical gravitational potential energy required for the steel ball to break the sample by the sample thickness.

Very small-angle neutron scattering (VSANS)

VSANS experiments were conducted at the VSANS diffractometer of the China Spallation Neutron Source (CSNS) in Dongguan, China. A minimum Q value of 0.0028 nm^{-1} was attained by converging twelve multi-slits into multiple narrow beams onto a high-resolution gas electron multiplier detector. The dried hydrogel samples were swollen in D_2O, and then encapsulated in sealed quartz sandwich cells (2 mm thickness) and positioned on a temperature-controlled stage. Raw sample data were processed to yield scattering profiles and absolute scattering intensities. D_2O solvent background was subtracted.

Low-field ^{1}H NMR measurements

Low-field ^{1}H NMR spectra were collected on an NMR analyzer (VTMR20-010V-I, Suzhou Niumai analytical instrument corporation) equipped with a hydrogen probe to measure the T_2 values of hydrogels. For measurements, hydrogels were placed in a sealed chromatography vial and tested in the sample chamber. Temperature-variable data were collected from 25 to 80 °C with 5 °C interval.

Temperature-variable IR measurements

FTIR spectra were collected on a Nicolet iS50 FTIR spectrometer in the transmission mode. For sample preparation, an appropriate amount of precursor solution was sandwiched between two CaF_2 tablets. In-situ polymerization was then employed to directly form the hydrogel film within this confined space. To prevent water loss during the subsequent heating process, the tablets were sealed with parafilm. For temperature-variable measurement, the sample was subjected to a heating ramp from 20 to 80 °C with an increment of 2 °C.

Perturbation-correlation moving window (PCMW)

All the temperature-variable IR spectra of P(AA_2Ca-*co*-AAm) hydrogel recorded during heating were employed to perform PCMW analysis. Raw data processing was carried out, and further correlation was calculated by the software 2D Shige, ver. 1.3 (©Shigeaki Morita, Kwansei Gakuin University, Japan, 2004-2005) with an appropriate window size ($2m + 1 = 11$). Finally, the contour maps were plotted using OriginPro 2024 program with red colors indicating positive intensities while blue colors the negative ones.

2D correlation spectroscopy (2DCOS)

All the temperature-variable FTIR spectra of P(AA_2Ca-*co*-AAm) hydrogel were used for performing 2D correlation analysis. 2D correlation analysis was carried out using the software 2D Shige, ver. 1.3 (©Shigeaki Morita, Kwansei Gakuin University, Japan, 2004-2005), and further plotted into contour maps by OriginPro 2024 program. In the contour maps, red colors are defined as positive intensities, while blue colors as negative ones.

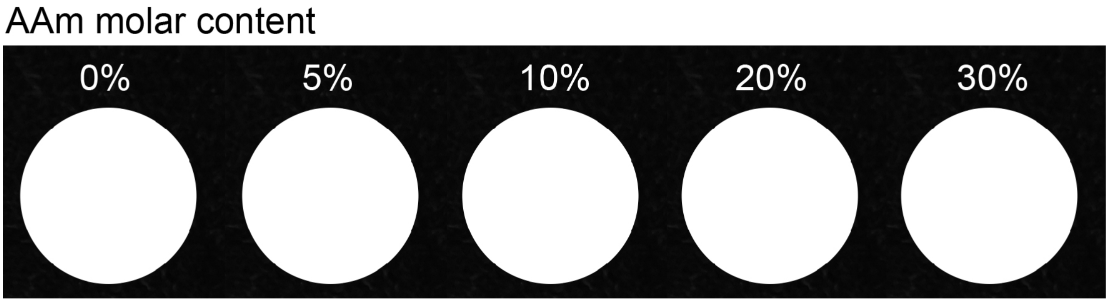

Fig. S1. Photos of P(AA_2Ca-*co*-AAm) hydrogels with increasing AAm molar contents. All the hydrogels appeared opaque.

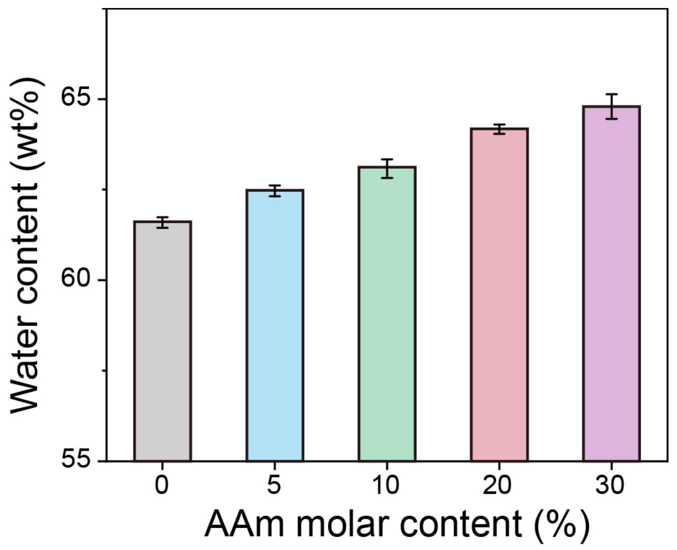

Fig. S2. Water contents of swollen P(AA_2Ca-*co*-AAm) hydrogels with increasing AAm contents at 20 °C. Increasing AAm content increased the water content of the resulting hydrogel.

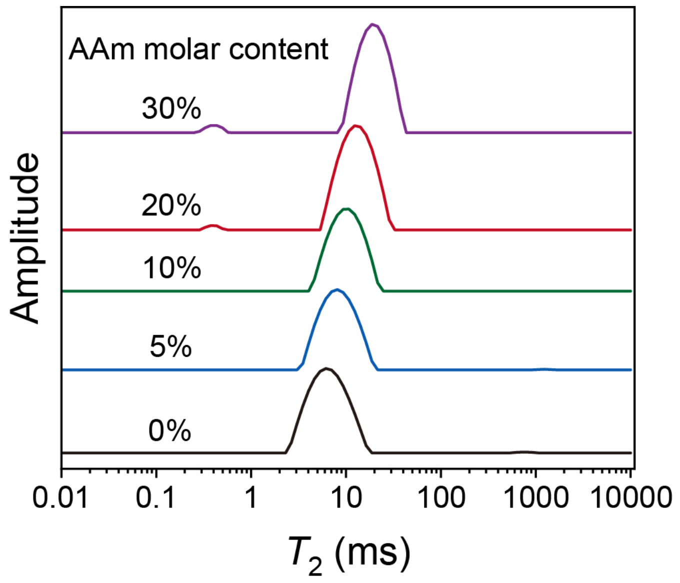

Fig. S3. Low-field ^1H NMR spectra of P(AA_2Ca-*co*-AAm) hydrogels with increasing AAm contents at 20 °C. The small T_2 peak at ~0.4 ms originated from PAAm chains. With increasing AAm contents, the water mobility increased as reflected by the shift of the main T_2 peak to higher relaxation times.

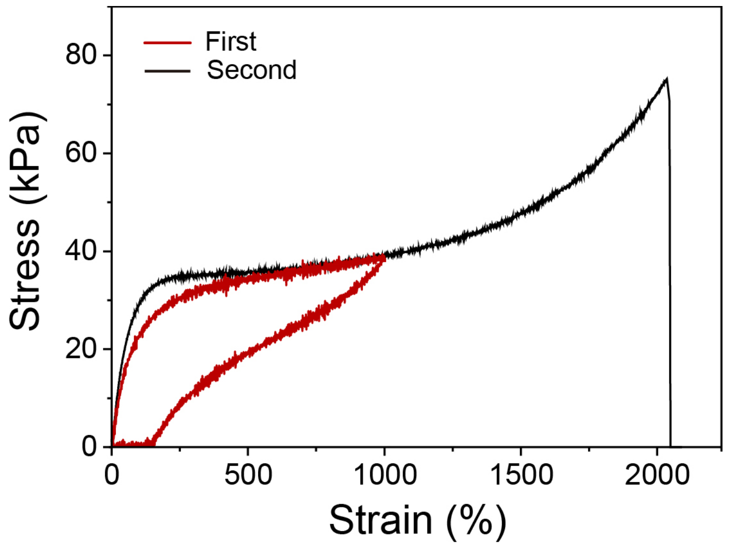

Fig. S4. Load-unloading (red) and secondary loading (black) curves of P(AA2Ca-*co*-AAm) hydrogel with 20% AAm at 20 °C. The almost coincident loading curves indicate the good elasticity of the hydrogel.

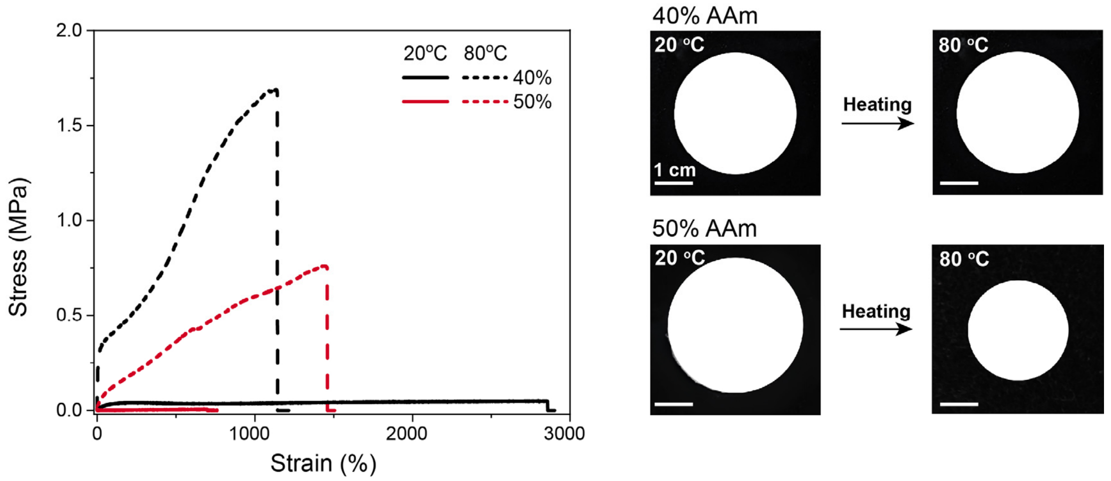

Fig. S5. Tensile stress-strain curves and photos of P(AA2Ca-*co*-AAm) hydrogels with 40% and 50% AAm at 20 and 80 °C, respectively. There are no obvious yielding points for these two hydrogels in the stiffened state. This indicates that the stiffened hydrogels did not effectively enter the glassy state, resulting in a reduced stiffening response. Moreover, significant heat-induced volume contraction was observed for the hydrogel with 50% AAm.

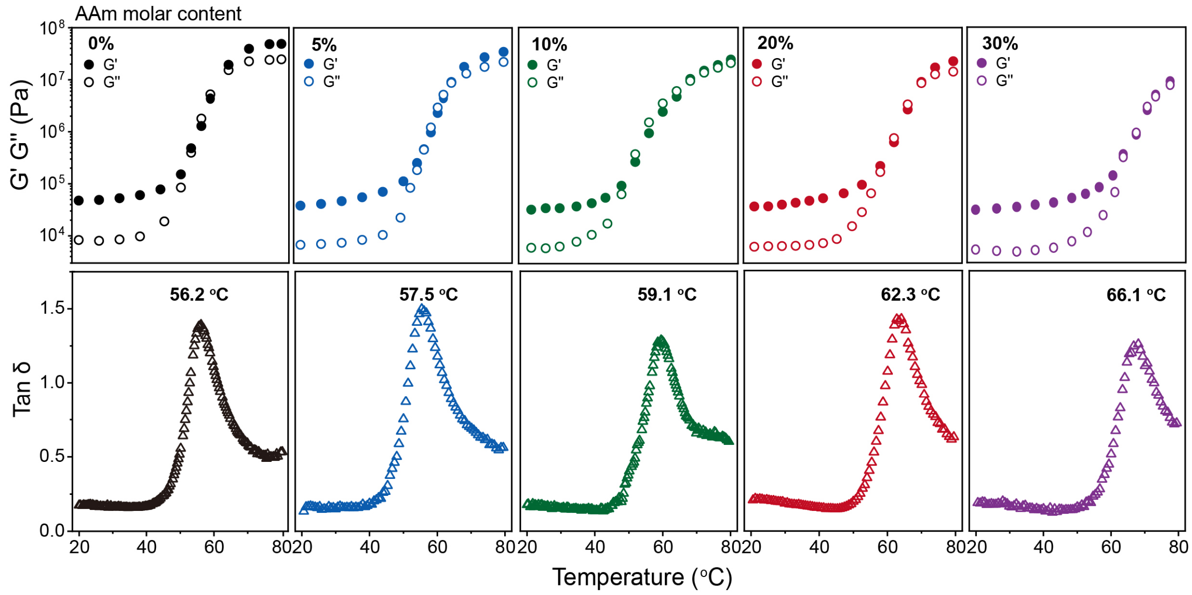

Fig. S6. Temperature-sweep rheological and corresponding tan δ curves of P(AA2Ca-*co*-AAm) hydrogels with increasing AAm molar contents. The tan δ peak temperatures were employed to determine their thermal-stiffening temperatures.

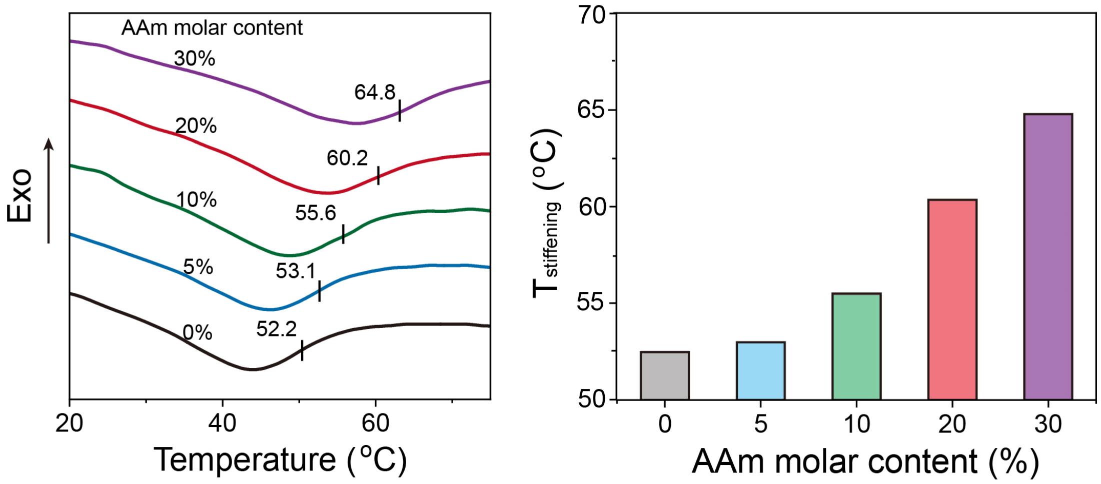

Fig. S7. DSC heating curves of P(AA2Ca-*co*-AAm) hydrogels with increasing AAm molar contents (heating rate: 5 °C min^{-1}). The stiffening temperatures read from DSC are slightly lower than those from rheological tan δ peaks.

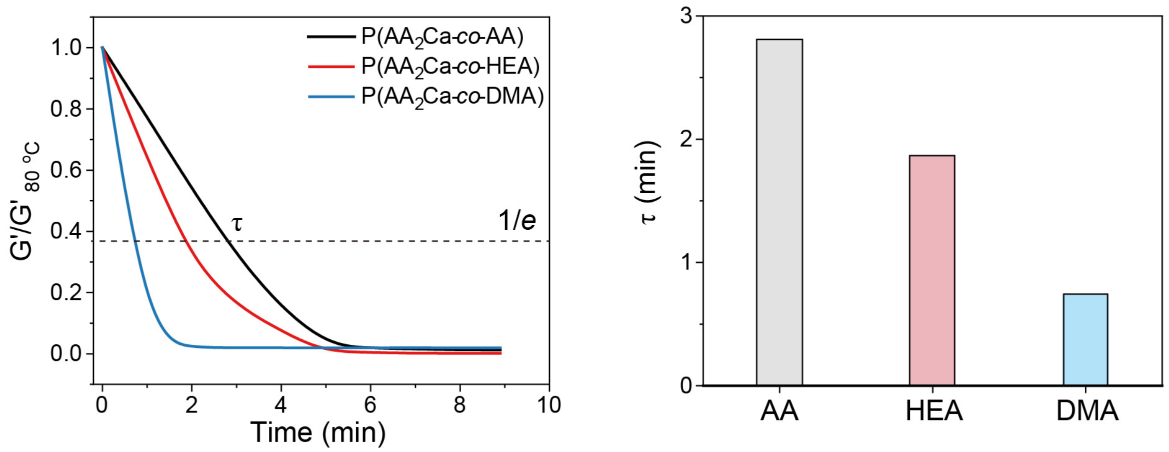

Fig. S8. Time-dependent storage moduli recovery and characteristic recovery times of P(AA_2Ca-*co*-AA), P(AA_2Ca-*co*-HEA), and P(AA_2Ca-*co*-DMA) hydrogels. All hydrogels were initially cured at 80 °C for 30 min and subsequently immersed in room-temperature water for monitoring. Compared to neat PAA_2Ca hydrogel, these three copolymer hydrogels all exhibited markedly reduced recovery times.

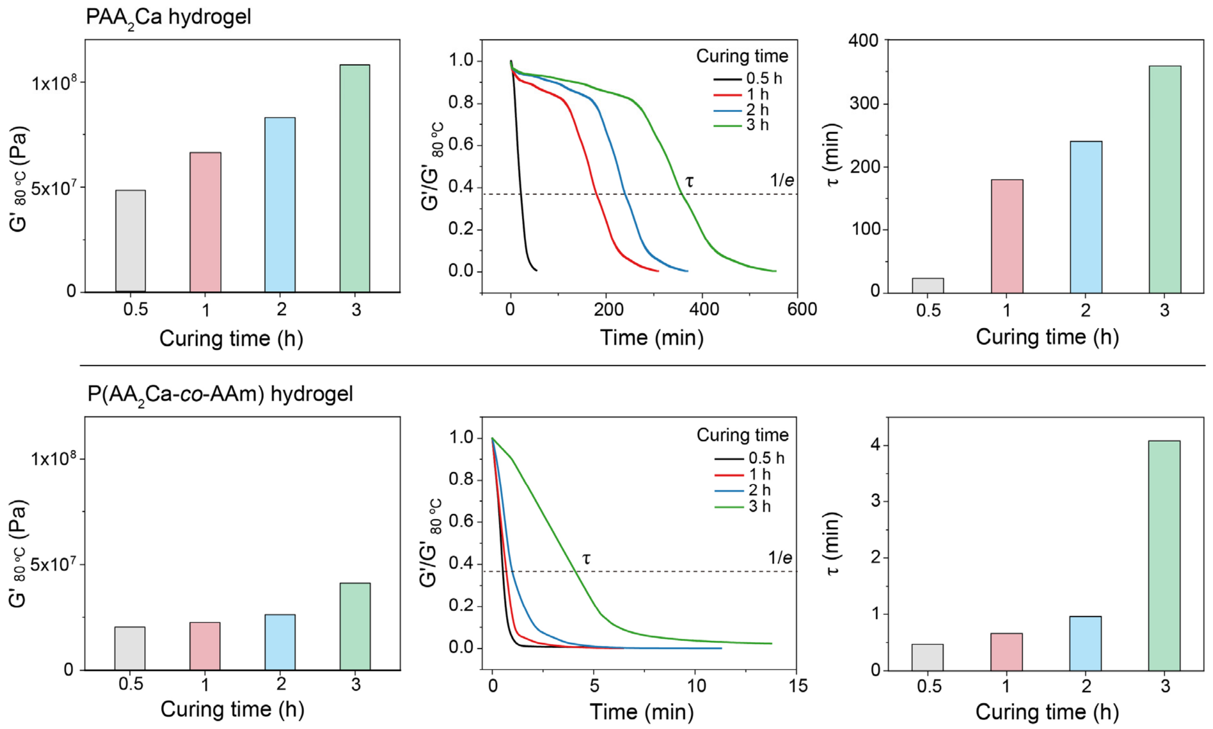

Fig. S9. Stiffened storage moduli, time-dependent moduli recovery, and characteristic recovery times of PAA_2Ca and P(AA_2Ca-*co*-AAm) hydrogels cured for various durations. The hydrogels were initially cured at 80 °C for different durations and subsequently immersed in room-temperature water for monitoring. The stiffened-state moduli of both hydrogels increased with longer curing times, indicating a greater degree of phase separation. Furthermore, increasing the curing time from 0.5 to 3 h dramatically increased the characteristic recovery time of the PAA_2Ca hydrogel, from 23 to 358 min. In contrast, the characteristic recovery time of the P(AA_2Ca-*co*-AAm) hydrogel remained consistently low (< 4 min) for curing times up to 3 h, significantly shorter than that of PAA_2Ca hydrogel. These findings demonstrate that incorporating hydrophilic AAm units substantially enhances the recovery behavior of these thermal-stiffening hydrogels.

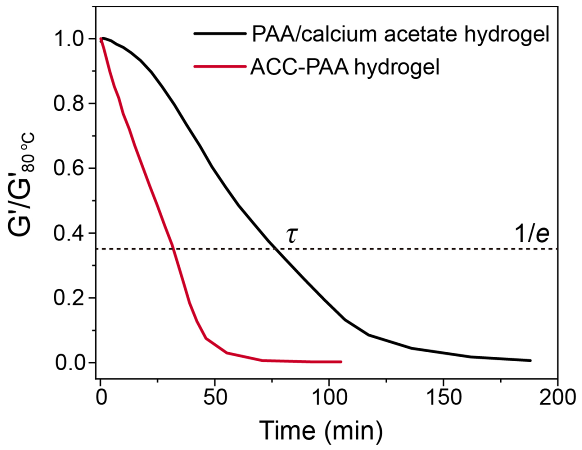

Fig. S10. Time-sweep storage modulus recovery of the stiffened PAA/calcium acetate and ACC-PAA hydrogels (cured at 80 ºC for 30 min). The characteristic recovery times of PAA/calcium acetate and ACC-PAA hydrogels were calculated to be 77 and 31 min, respectively.

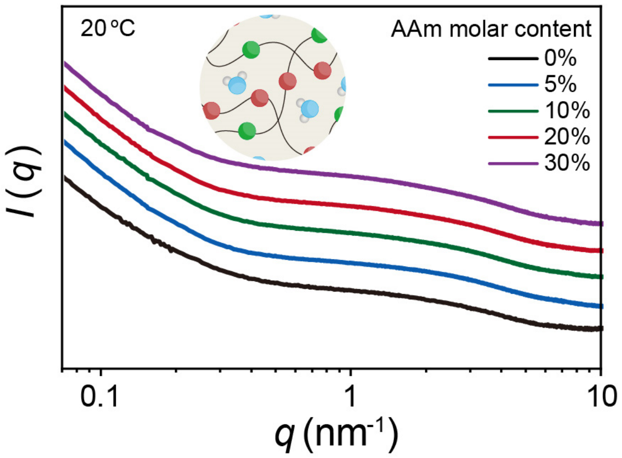

Fig. S11. Stacked SAXS profiles of P(AA_2Ca-*co*-AAm) hydrogels with increasing AAm contents at 20 ºC. $I(q)$ was plotted on a logarithmic scale. The scattering patterns of all hydrogels exhibited very similar characteristics. The calculated correlation length, reflecting the average distance between scattering entities, showed a rising trend with AAm content, ranging from 0.45 nm for 0% AAm (neat PAA_2Ca) to 0.64 nm for 30% AAm. This finding implies that incorporating AAm at room temperature led to a slight swelling of the gel while maintaining a similar network structure.

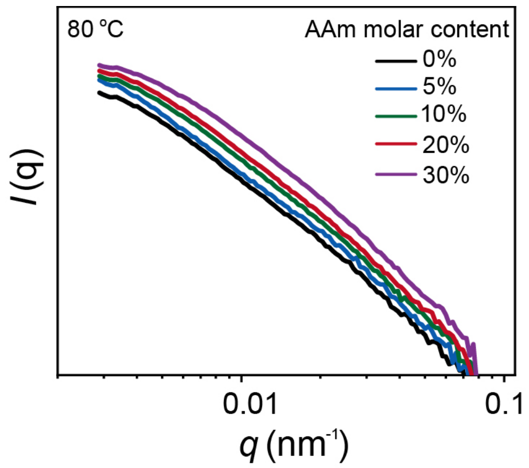

Fig. S12. Stacked VSANS profiles of P(AA$_2$Ca-*co*-AAm) hydrogels with increasing AAm contents at 80 °C. *I*(*q*) was plotted on a logarithmic scale. All the hydrogels showed a very similar phase-separated structure with a gyration radius (R_g) larger than 4.4 µm, corresponding to an average domain size of approximately 11 µm (2.58 R_g). This implies that the observed increase in topological entropy predominantly occurred at the nanoscale.

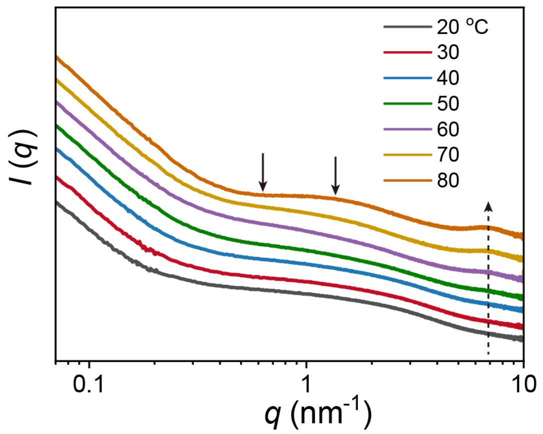

Fig. S13. Stacked temperature-dependent SAXS curves of P(AA$_2$Ca-*co*-AAm) hydrogel with 20% AAm. *I*(*q*) was plotted on a logarithmic scale. As the temperature increased, the peaks for collapsed PAA$_2$Ca chains at 7 nm^{-1} and for chain clustering at ~1.5 nm^{-1} both gradually emerged, characteristic for the stiffened polymer network. Moreover, significant changes can be observed starting from 60 °C, where a distinct plateau appeared in the intermediate *q* regime, indicating the loose cluster packing separated by AAm units.

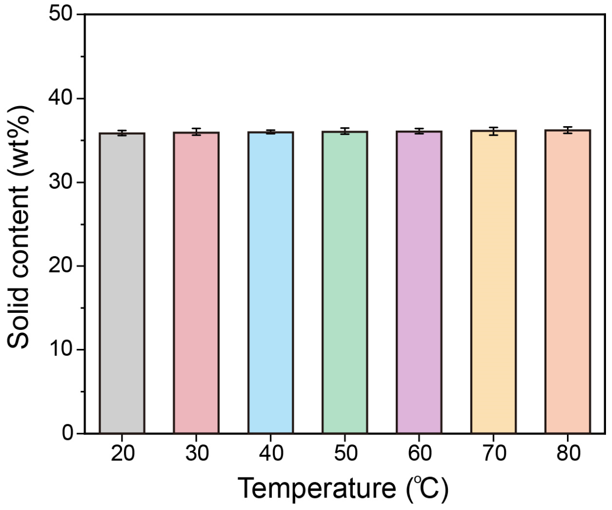

Fig. S14. Solid contents of P(AA_2Ca-*co*-AAm) hydrogel with 20% AAm at different temperatures. There were no apparent volume and weight changes in the thermal-stiffening process of the copolymer hydrogel.

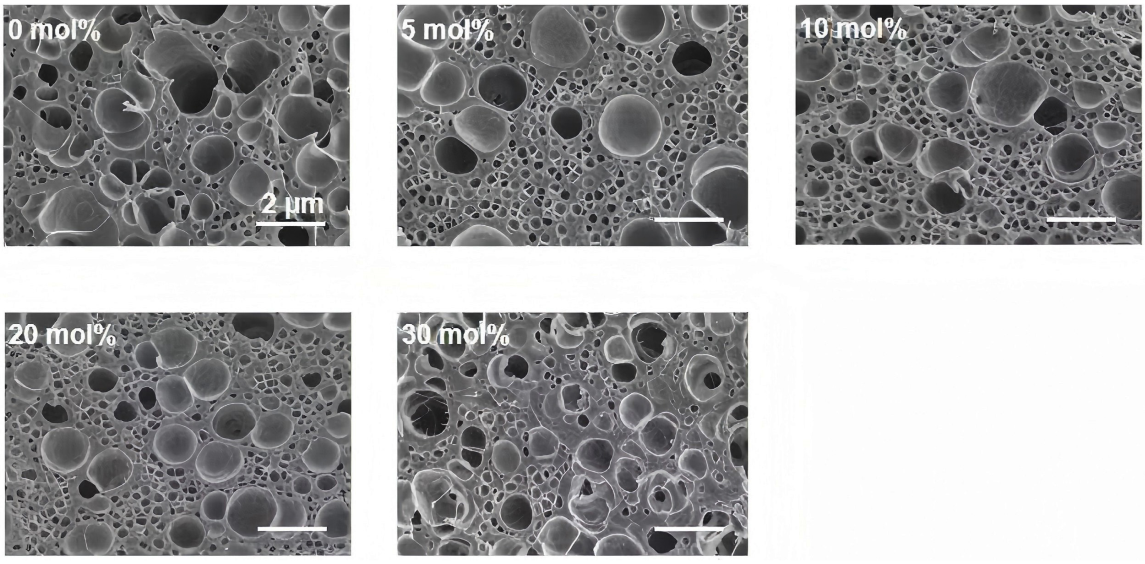

Fig. S15. SEM images of lyophilized P(AA_2Ca-*co*-AAm) hydrogels with increasing AAm contents. All the samples were prepared by pre-heating to 80 ºC and then quenched in liquid nitrogen. The images reveal no significant differences in morphology across different AAm contents, suggesting that the observed high-entropy structure arises primarily from the nanoassembly of thermal-stiffening clusters.

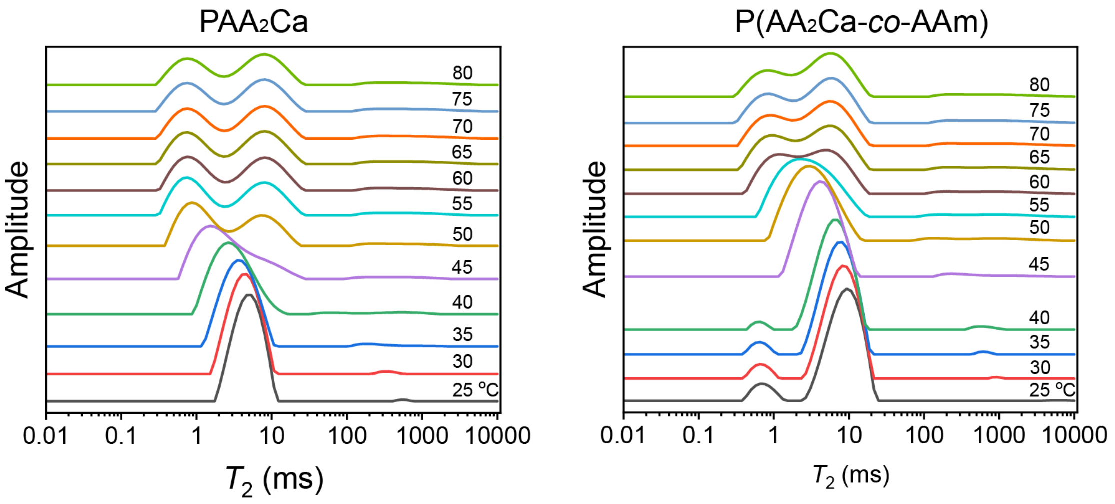

Fig. S16. Temperature-variable low-field ^1H NMR spectra of neat PAA_2Ca and P(AA_2Ca-*co*-AAm) hydrogels, respectively. At room temperature, both hydrogels presented a primary T_2 peak in the range of 6-10 ms, which is attributed to bound water associated with polymer chains. In comparison to neat PAA_2Ca hydrogel, the P(AA_2Ca-*co*-AAm) hydrogel demonstrated a reduced confinement of water molecules, owing to the incorporation of neutral AAm units. With increasing temperature, a peak shift towards lower T_2 values was observed, indicating enhanced spatial confinement of water molecules in conjunction with the reduced solubility of Ca^{2+}:COO^- complexes. Across the stiffening transition, both hydrogels exhibited peak splitting, transitioning from a homogenous bound water state to distinct populations of confined water at lower T_2 and intermediate water at higher T_2. This peak deconvolution signifies the onset of pronounced phase separation during the thermal-stiffening process.

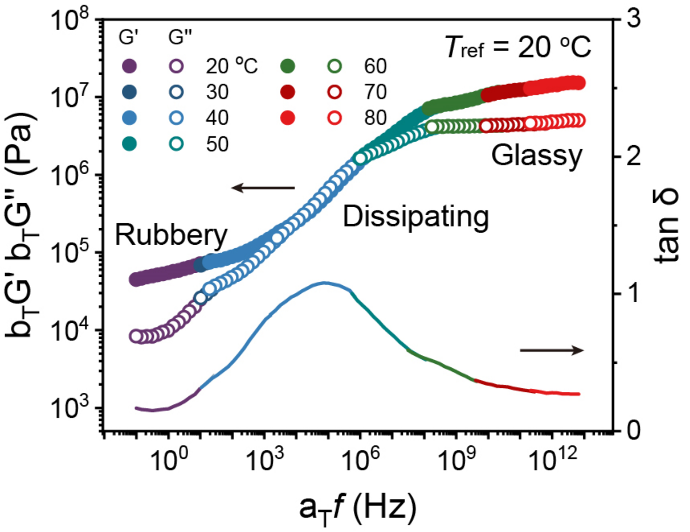

Fig. S17. Reverse time-temperature superposition rheological curves of neat PAA_2Ca hydrogel. 20 °C was chosen as the reference temperature. Neat PAA_2Ca hydrogel transitioned from rubbery to dissipating and glassy states with increasing temperatures or frequencies. At 80 °C, the tan δ value is about 0.3 in the stiffened state.

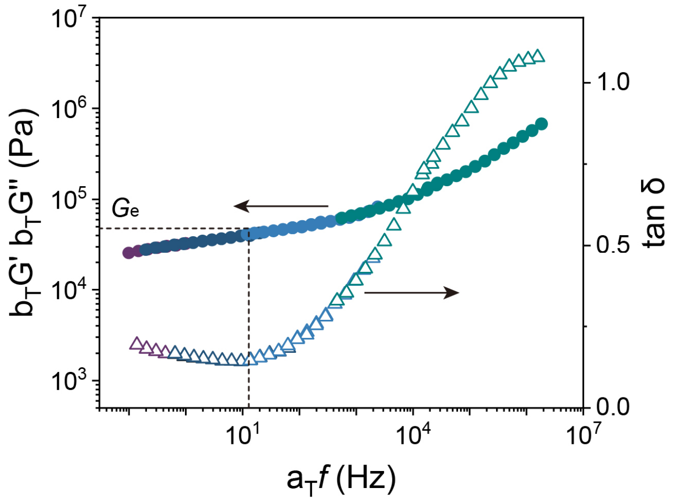

Fig. S18. The minimum tan δ method for calculating the number-average molar mass of the entanglement strands (M_e) in the P(AA_2Ca-*co*-AAm) hydrogel. The entanglement plateau modulus (G_e) was read to be 4.2×10^4 Pa. M_e was calculated to be 8.2×10^4 g mol^{-1} according to the equation $M_e = \rho RT/G_e$, where ρ is the density, R the universal gas constant, and T the absolute temperature [S1].

Table S1. Multiplication results of the signs of each cross-peak in 2DCOS synchronous and asynchronous spectra of P(AA$_2$Ca-*co*-AAm) hydrogel (Fig. 4f).

1538	+	+	+	+	+	
1562	+	+	+	+		
1596	+	+	+			
1641	-	+				
1650	-					
1683						
	1683	1650	1641	1596	1562	1538

As a mathematical method, the basic principle of 2DCOS was first proposed by Isao Noda [S2, 3]. Enhanced spectral resolution can be achieved by 2DCOS by expanding the original spectral information along the second dimension, allowing for the extraction of additional subtle information about molecular motions or conformational changes. 2DCOS includes two kinds of correlation maps. Synchronous spectrum reflects the simultaneous change of two wavenumbers, and asynchronous spectrum can significantly enhance spectral resolution. In synchronous spectrum, automatic peaks occur only along the diagonal, while cross-peaks can occur in both synchronous and asynchronous spectra.

The judging rule of the sequence can be summarized as Noda's rule: if the multiplication of the signs of cross-peaks (v_1, v_2, and assume $v_1 > v_2$) in synchronous and asynchronous spectra is positive, the change at v_1 occurs prior to v_2, and vice versa [S4]. In Table S1, "+" means the same signs in synchronous and asynchronous spectra, while "-" means different signs. According to Noda's rule, the final specific order for P(AA$_2$Ca-*co*-AAm) hydrogel during heating is given as follows (→ means prior to or earlier than): 1650 → 1641 → 1683 → 1596 → 1562 → 1562 → 1538 cm^{-1}, i.e., δ(O-H) (intermediate water) → v(C=O) (amide I, PAAm) → δ(O-H) (confined water) → v_{as}(COO$^-$) (free) → v_{as} (COO$^-$) (weakly coordinated with Ca^{2+}) → v_{as}(COO$^-$) (strongly coordinated with Ca^{2+}).

REFERENCES

S1. Daniel WFM, Burdyńska J, Vatankhah-Varnoosfaderani M *et al.* Solvent-free, supersoft and superelastic bottlebrush melts and networks. *Nat Mater* 2016; **15**: 183-9.

S2. Noda I. Two-dimensional infrared (2D IR) spectroscopy: theory and applications. *Appl Spectrosc* 1990; **44**: 550-61.

S3. Noda I. Two-dimensional infrared spectroscopy. *J Am Chem Soc* 1989; **111**: 8116-8.

S4. Sun S, Wu P. Spectral insights into microdynamics of thermoresponsive polymers from the perspective of two-dimensional correlation spectroscopy. *Chin J Polym Sci* 2017; **35**: 700-12.
